# Regulatory RNA binding proteins contribute to the transcriptome-wide splicing alterations in human cellular senescence

**DOI:** 10.18632/aging.101485

**Published:** 2018-06-24

**Authors:** Qiongye Dong, Lei Wei, Michael Q. Zhang, Xiaowo Wang

**Affiliations:** 1 Ministry of Education Key Laboratory of Bioinformatics, Center for Synthetic and Systems Biology, Department of Automation, Tsinghua University, Beijing 100084, China; 2 Bioinformatics Division, Beijing National Research Center for Information Science and Technology, Beijing 100084, China; 3 Department of Basic Medical Sciences, School of Medicine, Tsinghua University, Beijing 100084, China; 4 Department of Biological Sciences, Center for Systems Biology, The University of Texas, Richardson, TX 75080, USA

**Keywords:** cellular senescence, alternative splicing, RNA binding proteins, splicing regulation, RNA-seq

## Abstract

Dysregulation of mRNA splicing has been observed in certain cellular senescence process. However, the common splicing alterations on the whole transcriptome shared by various types of senescence are poorly understood. In order to systematically identify senescence-associated transcriptomic changes in genome-wide scale, we collected RNA sequencing datasets of different human cell types with a variety of senescence-inducing methods from public databases and performed meta-analysis. First, we discovered that a group of RNA binding proteins were consistently down-regulated in diverse senescent samples and identified 406 senescence-associated common differential splicing events. Then, eight differentially expressed RNA binding proteins were predicted to regulate these senescence-associated splicing alterations through an enrichment analysis of their RNA binding information, including motif scanning and enhanced cross-linking immunoprecipitation data. In addition, we constructed the splicing regulatory modules that might contribute to senescence-associated biological processes. Finally, it was confirmed that knockdown of the predicted senescence-associated potential splicing regulators through shRNAs in HepG2 cell line could result in senescence-like splicing changes. Taken together, our work demonstrated a broad range of common changes in mRNA splicing switches and detected their central regulatory RNA binding proteins during senescence. These findings would help to better understand the coordinating splicing alterations in cellular senescence.

## Introduction

Cellular senescence (CS), a stable cell-cycle arrest, was initially observed that normal fibroblasts had a limited ability to proliferate [1]. This type of senescence is known as “replicative senescence” or “Hayflick limit”. Various external signals can also trigger CS, such as oncogene-induced senescence (OIS) and stress-induced premature senescence (SIPS) [2]. The senescent program has been widely recognized as a barrier to cancer due to its permanent growth arrest [3]. On the other hand, the accumulation of senescent cells might contribute to organismal ageing, and the clearance of senescent cells could delay ageing-associated phenotypes and extend healthy lifespan [4,5]. Aside from cell cycle arrest, large-scale changes occurred in senescent cells at the cellular and molecular levels, including gene expression changes, as well as splicing alterations [6].

Alternative splicing (AS) is a post-transcriptional process, during which a single gene can produce multiple different protein isoforms. It greatly increases the protein biodiversity. Dysregulations of mRNA splicing are emerging to be discovered as important players in organismal ageing, cellular senescence and ageing-related degenerative diseases [7]. For example, the upregulation of p44, a short isoform of TP53, could activate the insulin-like growth factor (IGF) signaling pathway in *C. elegans, D. melanogaster* and mice, and would accelerate ageing and growth arrest [8]. ING1 gene encoded tumor suppressor proteins that affected cell growth, apoptosis and response to DNA damage [9]. It was reported that the ratio between its two splicing isoforms, ING1a and ING1b, increased in CS. Ectopic overexpression of ING1a in young cells would elicit senescence-associated phenotypes [9,10]. The premature ageing disease Hutchinson–Gilford progeria syndrome was caused by a single-point mutation, which would lead to the mis-splicing of nuclear lamin A/C (LMNA) gene and a remarkable increase of progerin proteins [11].

Proper expression of the splicing regulatory RNA-binding protein (RBP) genes is necessary for the precise AS program [12]. The RBPs may interact with other proteins, as well as RNAs, and form the ribonucleoprotein complexes (RNPs) that mediate pre-mRNA splicing of different exons or splice sites [13,14]. It was found that isoform ratios and gene expressions of RBPs changed with ageing. Harries et al. revealed that genes with expression alterations in advanced ageing were enriched in gene sets associated with mRNA splicing and other post-transcriptional pathways [15]. Holly et al. demonstrated that the expressions of key splicing RBPs were associated with age by analyzing blood samples from two human populations. This result was validated in human senescent fibroblasts and endothelial cells [16]. Furthermore, Lee et al. also revealed that certain post-transcriptional alterations were associated with lifespan through a research on six mouse strains of different longevities. They inferred that correct regulations of AS might enhance lifespan in mice, and even in human [17].

Some efforts have been devoted to the study of transcriptome in CS process. Recently, an integrative analysis of RNA-seq data focused on the heterogeneity across various types of senescent cells, and identified core gene signatures that were commonly differentially expressed in diverse senescent samples [6]. Likewise, it is necessary to characterize the consistent splicing alterations on the whole transcriptome and infer their potential splicing regulatory RBPs, which is poorly understood in cellular senescence. Extensive splicing changes were found in replicative senescence through splicing-sensitive microarray [18]. However, to the best of our knowledge, no systematic investigation of transcriptome-wide splicing alterations in CS has been reported before.

In this study, we integrated publicly available RNA sequencing datasets of diverse human senescent samples and found that down-regulated genes in cellular senescence were significantly enriched with RBP genes, especially the major regulators in the biological processes associated with mRNA splicing. We identified common differential splicing events in CS across different induction methods and different cell types. Further investigations on these CS-associated differential splicing events through combining their RNA binding characteristics discovered that they were mainly regulated by these eight splicing regulatory RBPs: SRSF1, SRSF7, QKI, RBFOX2, PTBP1, HNRNPK, HNRNPM and HNRNPUL1. To clarify how these identified splicing RBPs contributed to the alternative splicing changes in cellular senescence, we inferred a splicing regulatory network through the RNA binding information. Finally, the roles in splicing regulation of these predicted RBPs in CS were validated through analyzing RNA-seq data with single-gene knockdown experiments collected from the ENCODE database. Taken together, our study provided a comprehensive understanding of the mechanism of the alternative splicing events and their regulatory relationships during CS from a global transcriptomic view. Our investigation highlighted the potential key CS-associated splicing regulatory RBPs through an integrative approach.

## RESULTS

### Down-regulated genes in cellular senescence are enriched with RBPs

In order to identify genes that were consistently differentially expressed in diverse CS samples from different cell types with different induction methods compared with the samples in the growing state, we collected publicly available RNA sequencing data of human CS experiments, including five cell types (IMR90, WI38, HFF, BJ and astrocytes) and four senescent induction approaches (replicative senescence, and senescence respectively induced by RAS, drug and high-oxidation) (details shown in Table S1). Consistently differentially expressed genes in all these senescent experiments were identified through meta-analysis, including 1082 up-regulated genes and 1073 down-regulated genes (Table S2-3, Figure S1A).

**Figure 1:**
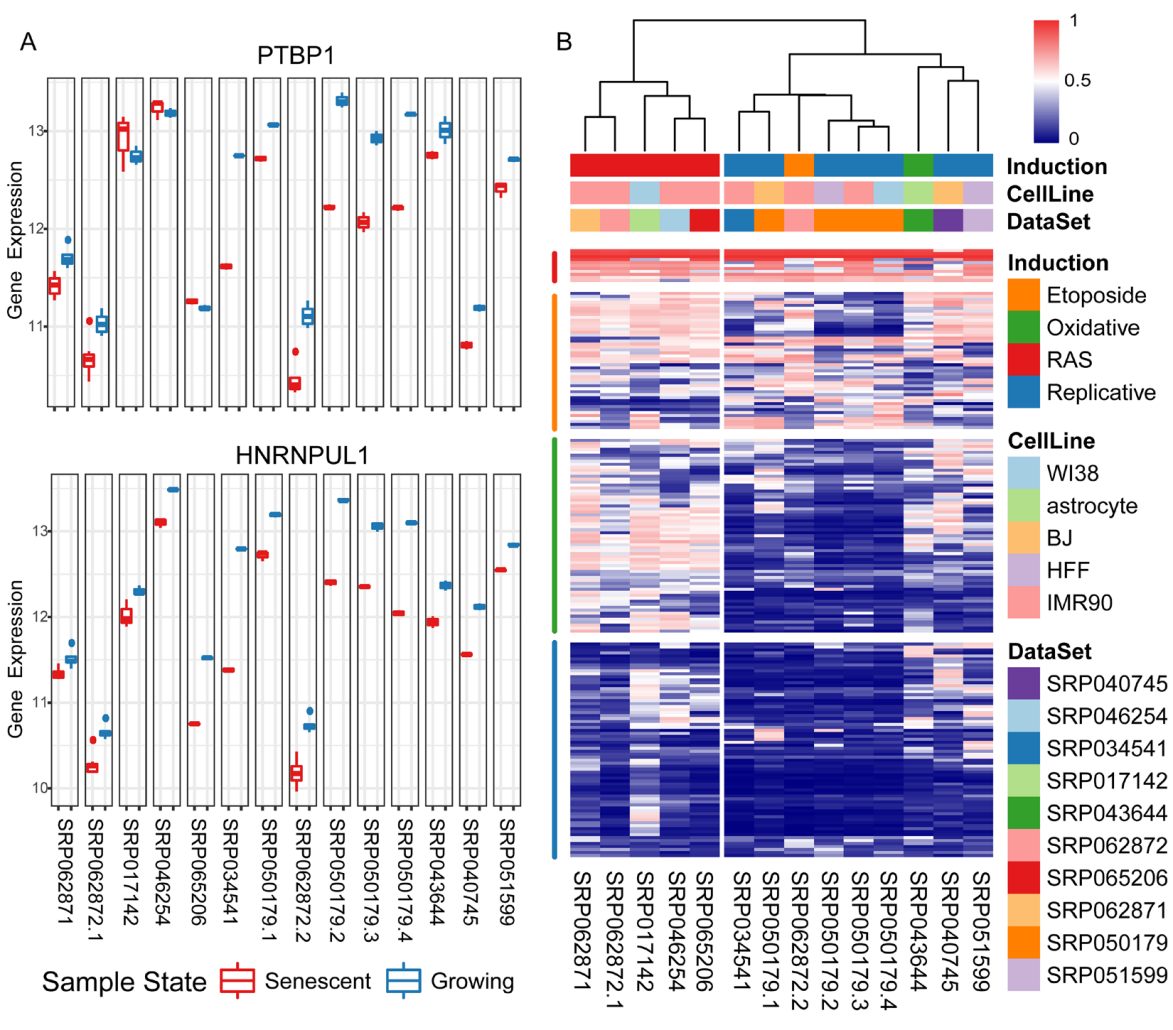
**Differential expression levels of RNA binding proteins (RBPs) in senescent samples compared with growing ones.** (**A**) Taking two consistently down-regulated RBPs as examples, their gene expression levels (y-axis: log2 read counts) of senescent samples compared with growing ones in fourteen experiments (x-axis). (**B**) Heatmap using a rank-based visualization method to present the differential expression levels of collected RBPs in all experiments respectively. Each column represents an experiment and each row represents one gene. A normalized rank transform is performed on each individual experiment by sorting the p-values from the most down-regulated with the lowest 0 (blue) to the most up-regulated with the highest 1 (red).

Functional roles of the differentially expressed genes were revealed via Gene Ontology (GO) enrichment analysis (Figure S1C, Table S4). As expected, top enriched Biological Process (BP) GO terms were associated with ageing or cellular senescence. Down-regulated genes were enriched in the processes of cell division, DNA replication, DNA repair, etc., while up-regulated genes were enriched in the processes associated with proton transport, autophagy, etc. Interestingly, biological processes associated with mRNA splicing were listed among the top significantly enriched down-regulated ones. RBP genes, major controllers in regulating mRNA post-transcription, showed aberrantly expression in senescent samples compared with growing ones. Among the 192 RBPs, collected in this study (Materials and Methods), 22.1% were significantly down-regulated in senescent samples (p-value = 2.53e-19, odds ratio = 6.629) (Table S5). Taking PTBP1 and HNRNPUL1 as examples shown in Figure 1A, their gene expression levels are consistently down-regulated in senescent samples in multiple experiments.

Furthermore, in order to infer the RBPs’ expression patterns in different types of CS, we performed clustering analysis of their normalized differential expression levels in fourteen CS experiments on both the experiment and RBP dimensions, respectively (Figure 1B). All experiments were separated into two clusters according to the senescence-inducing methods: replicative senescence (left), senescence induced by other methods (right). The collected 192 RBPs could be partitioned into four major groups according to their differentially expressed levels in fourteen experiments: common up-regulated (red), no regular patterns and no significant alterations in either of two clusters (orange), only significantly down-regulated in replicative senescence but not in other inducing condition (green), and consistently down-expressed in diverse experiments (blue). In summary, a significant proportion of RBPs were consistently down-regulated in diverse senescent cells (Figure S2A), and the common differentially expressed RBPs were treated as potential splicing regulators during CS.

### Genes with differential splicing events in CS function in ageing-associated GOs

We hypothesized that alterations on RBP gene expressions might cause alternative isoform changes during CS. To fully characterize the AS program difference between senescent and growing samples, we applied rMATS [19] on each dataset to identify differential splicing events and performed meta-analysis to find the common ones, which were defined as CS-associated differential splicing events. The number of splicing events varied among these experiments, which might be affected by read length and sequencing depth (Table S6).

**Figure 2:**
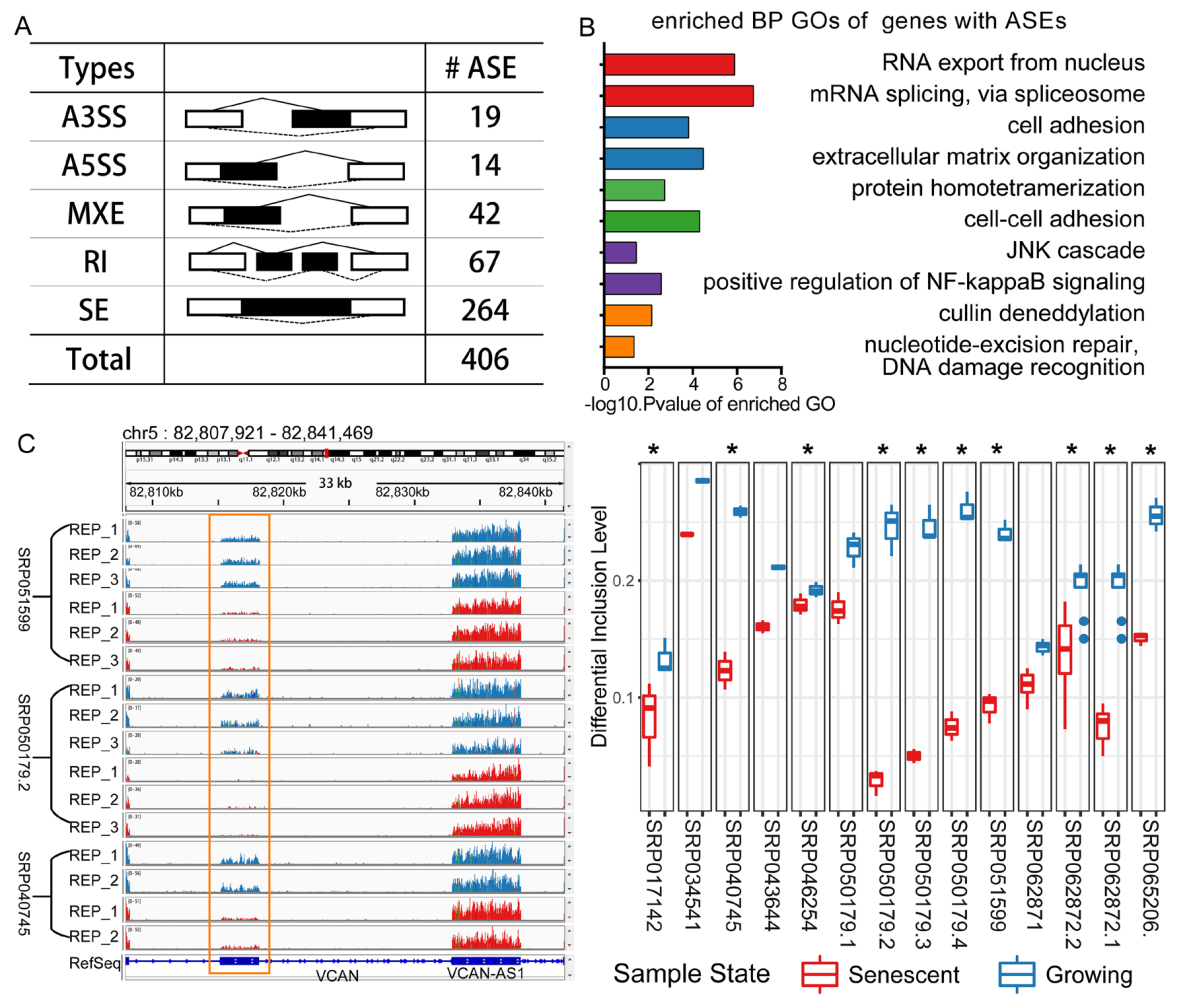
**Differential splicing analysis in cellular senescence.** (**A**) Statistics of the differential splicing events from the integrating result. (Abbreviations: SE: skipped exon; A5SS: Alternative 5’ splice site; A3SS: Alternative 3’ splice site; MXE: Mutually exclusive exon; RI: Retained intron). (**B**) GO enrichment analysis for the genes with CS-associated differential splicing events. (**C**) An example of one alternative splicing event of gene VCAN. VCAN is located on Chromosome 5 and this event is a MXE type, and two mutually exclusive exons separately locate from 82815167 to 82818128, 82832825 to 82838087. This MXE event is shown in RNA-seq read coverage plot (left: showing three experiments) in the genome browser and percent spliced in (PSI) boxplot (right: * representing differential spliced in this experiment).

We identified 406 CS-associated common differential splicing events (Table S7, Figure 2A). The majority of splicing patterns were events of skipped exons. One differential splicing event of VCAN is shown as an example in Figure 2C. This gene was reported spliced in ageing and cellular senescence [15,16]. The detected differentially spliced genes participated in functional processes, including mRNA splicing, extracellular matrix organization, positive regulation of NF-kappaB signaling pathway, etc. (Figure 2B). These processes were discovered associated with cellular senescence or ageing [3,20,21]. In order to infer the potential associations between the splicing events and phenotypes, we mapped known single nucleotide variants (SNVs) to the CS-associated splicing event regions, and identified 1071 SNVs with phenotypic annotation in NCBI ClinVar database [22] (Table S10). These genomic variants were assigned to 50 genes, many of which were associated with degenerative disorders (Table 1). For example, Hutchinson-Gilford progeria syndrome (HGPS)-associated SNVs in LMNA, a well-known spliced gene, were found within the CS-associated splicing event regions. In summary, these observations suggested that the identified common splicing alterations in diverse senescent samples might play important roles in the CS-related processes.

**Table 1:** Examples of genes with differential splicing events that were annotated with degenerative diseases associated SNVs.

**GENE SYMBOL**	**AS TYPES**	**ASSOCIATED DISEASES**
**ASPM**	SE	Primary Microcephaly
**LAMP2**	SE	Danon Disease
**COL6A3**	SE; MXE	Collagen VI-related myopathy
**LMNA**	RI	Hutchinson-Gilford syndrome, Cardiovascular phenotype
**TPM1**	SE; MXE	Dilated cardiomyopathy, Hypertrophic cardiomyopathy
**VCAN**	SE; MXE	Wagner syndrome, Vitreoretinopathy

### Eight splicing regulatory RBPs were identified as key senescence AS regulators

**Figure 3:**
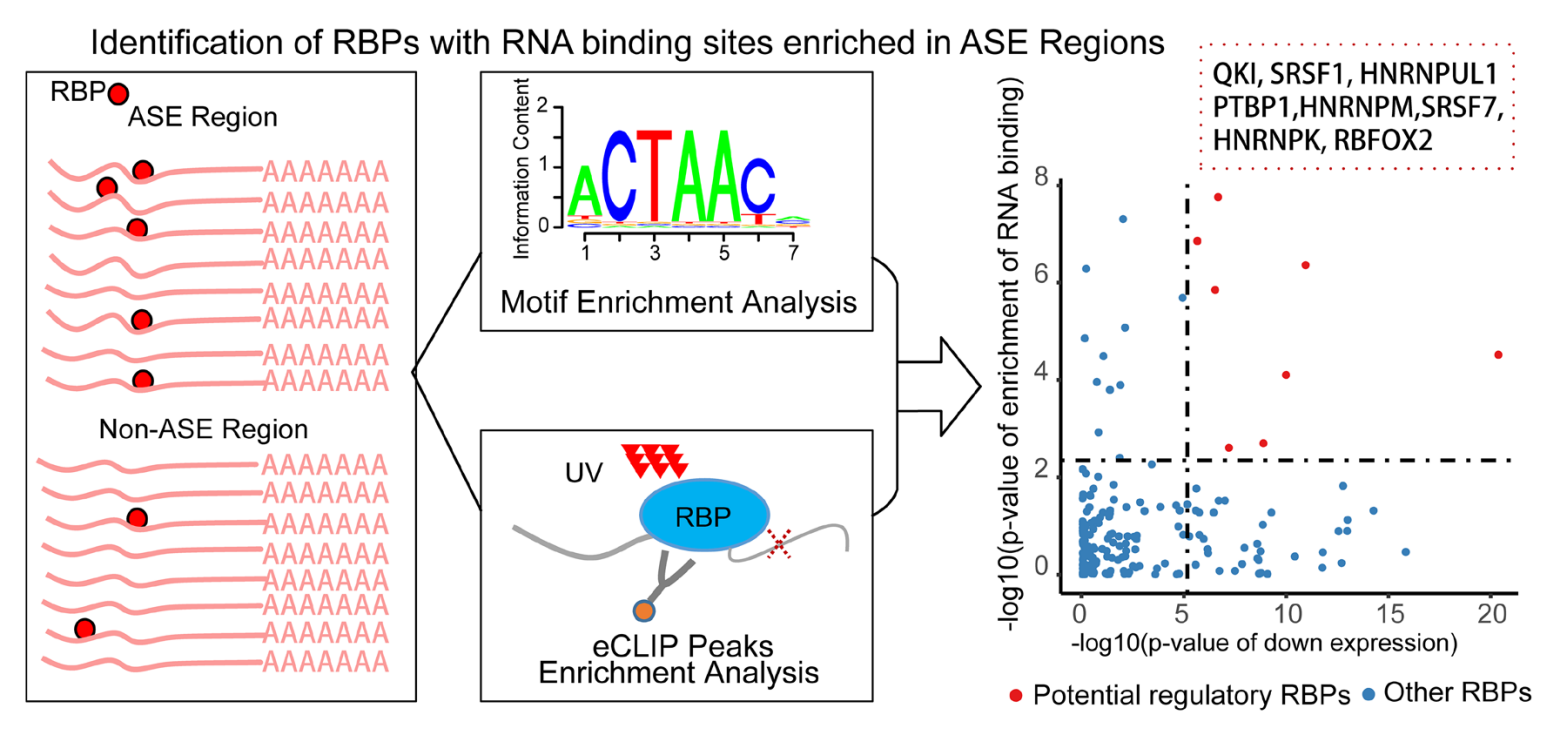
**Pipeline of identifying RNA binding proteins (RBPs) regulating the senescence-associated alternative splicing events.** For each RBP, the enrichment of RNA binding information of the Alternative Splicing Event (ASE) regions compared with the non-ASE regions through combining the methods of motif scanning and eCLIP peaks. Only the differentially expressed RBPs with significantly enriched RNA binding information were defined as the potential senescence-associated regulators (right scatter plot). x-axis is the p-value of down-regulations of the RBPs, y axis is the combined enrichment p-value. Red point is the detected eight splicing RBPs.

To predict the candidate RBPs that regulate differential splicing in CS, we performed an enrichment analysis of RBPs’ RNA binding sites around CS-associated differential splicing exon regions. Both approaches of scanning known RNA binding motifs [23] and the significant peaks of the eCLIP data [24] were applied to infer the RBPs’ RNA binding information (Figure 3, Table S8, Materials and Methods). Twenty RBPs were identified to have significant enrichment in RNA binding sites within the CS-associated differential splicing event regions. Only eight of these RBPs were significantly differentially expressed in diverse senescent samples, and all of them were consistently down regulated. These eight RBPs, namely, SRSF1, SRSF7, QKI, RBFOX2, PTBP1, HNRNPK, HNRNPM and HNRNPUL1, were predicted as the potential key AS regulators in cellular senescence.

### Identifying cellular senescence-associated splicing regulatory modules

**Figure 4:**
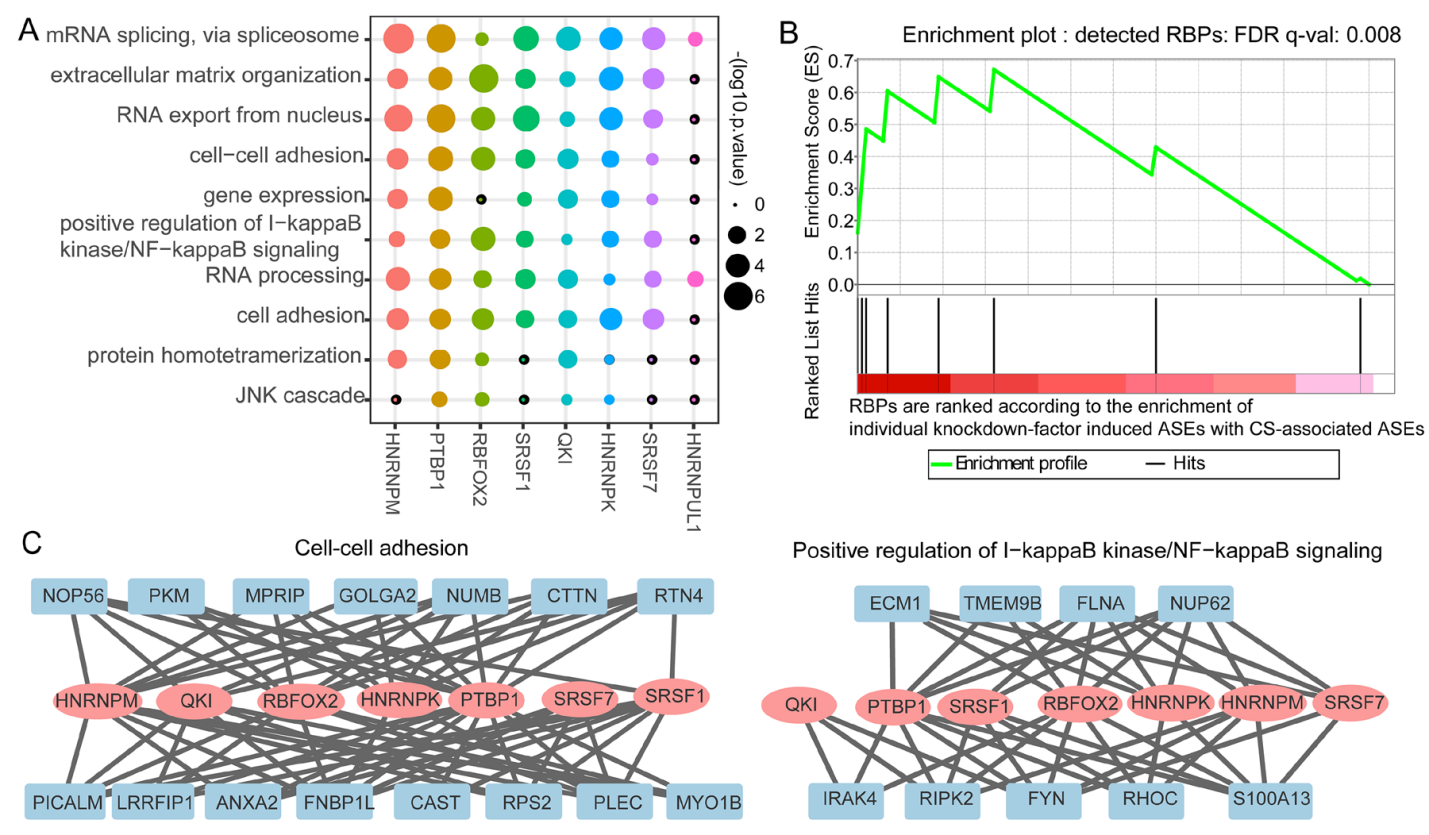
**Validation of identified potential splicing regulatory roles of RNA binding proteins’ (RBPs) in cellular senescence (CS)-associated splicing events and their regulatory modules.** (**A**) GO enrichment result of the identified candidate regulatory RBPs’ targeting genes. The size of the dot represents the log10 the enrichment p-values. (**B**) Gene set enrichment analysis (GSEA) plot for the detected eight splicing RBPs and the pre-ranked RBPs. The Y-axis gives the enrichment score for the enrichment of single factor knockdown-induced ASEs with CS-associated ASEs in the top panel. The X-axis refers to the rank of collected RBPs according to the enrichment. Each vertical line in the bottom panel of the ﬁgure refers to hit position of the eight splicing RBPs in the ranked list. HNRNPK, SRSF1 and QKI were identified as the top three enriched RBPs. (**C**) NF-kappa B signaling (left) and cell-cell adhesion (right) pathways intensively regulated by the CS-associated RBPs.

We assigned the differential splicing events to each identified potential senescence splicing regulatory RBP to build the splicing regulatory network. The role of each RBP was revealed through GO enrichment analysis of its targeting genes with differential splicing events. The splicing network uncovered that these RBPs controlled widespread splicing changes and affected a number of genes known to participate in each biological process during senescence (Figure 4A). For example, PTBP1 and RBFOX2 were predicted as the main splicing regulators in the process of cell-cell adhesion. It was discovered that reduction of PTBP1 and PTBP2 induced glioma cells to decrease proliferation and migration and to increase cell adhesion [25]. In addition, the splicing alterations involved in the NF-kappaB signaling pathway were predicted to be mainly targeted by RBFOX2 (Figure 4A and 4C). Taken together, the splicing regulatory network unveiled the potential functional roles for the identified regulatory RBPs in CS.

### Knocking down splicing regulatory RBPs induced CS-associated splicing events

To further validate the potential regulatory roles of these detected CS-associated splicing regulatory RBPs, we compared the overlap between differential splicing events identified from RBP knockdown experiments and the CS-associated differential splicing events. First, we collected 121 groups of individual RBP knockdown RNA-seq data of HepG2 cell line from ENCODE project [26]. Then, we identified the differential splicing profiles upon single RBP knockdown and compared the overlap between the RBP knockdown-induced splicing alterations with the 406 CS-associated differential splicing events. RBPs were ranked according to their overlap enrichment statistics for 121 RBPs. Finally, Gene Set Enrichment Analysis (GSEA) [27] result showed that eight predicted CS-associated splicing regulatory RBPs were among the top-ranked RBPs (Figure 4B, Table S9). It should be noted that, the most top three enriched RBPs, namely, QKI, SRSF1 and HNRNPK, were among our predicted CS-associated splicing controllers.

All of QKI, SRSF1 and HNRNPK played critical roles in CS-associated phenotypes with multiple evidence. Quaking (QKI) belongs to STAR (Signal Transduction and Activation of RNA) protein family. Its dysregulation contributed to many genetic diseases, such as increasing injury in diabetic heart and schizophrenia [28,29]. QKI was detected to mediate the alternative splicing of the Histone Variant MacroH2A1 [30], one of whose splicing isoform microH2A1.1 was found to regulate the SASP genes during the process of oncogene-induced senescence [31]. The second example is SRSF1 (serine/arginine-rich splicing factor). Recent research demonstrated its regulatory role in cell proliferation and migration. Its overexpression enhanced the proliferation of vascular smooth muscle cells (VSMCs), while its knockdown suppressed VSMCs’ growth [32]. Telomere shortening, as a result of replication, was a marker of replicative senescence and a senescence-inducing approach [33,34]. Meanwhile, it was discovered that homologues of the human hnRNP K in yeast could maintain the telomere length and the structural and functional organization of telomeric chromatin [35].

## DISCUSSION

Increasing evidence suggests that dysregulation of splicing factors and the production of key splice variants may attribute to cellular senescence or ageing-associated phenotypes [7]. However, a global transcriptomic landscape of AS program and the splicing regulatory factors are still largely unknown in CS. The rapid accumulation of RNA-seq data in public databases makes it possible to perform meta-analysis on transcriptomes across studies related with CS.

In order to identify consistent alterations in cellular senescence, we collected multiple RNA-seq datasets, and performed an integrative analysis of differential expression and alternative splicing. Regardless of their heterogeneous transcriptomes, we observed that a significant proportion of RBPs were consistently down-regulated in human senescent cells. This kind of expression changes of spliceosomal components and splicing regulatory factors might alter splicing profiles during CS. We uncovered consistent senescence-associated splicing patterns, detected corresponding regulatory RBPs through enrichment analysis of RNA binding sites in differential splicing event regions and predicted senescence-associated splicing regulatory modules.

Our work detected 406 consistently CS-associated differential splicing events in various types of senescent experiments. A number of known SNVs around these splicing regions were associated with degenerative disorders (Table 1, Table S10). For example, mutations of LMNA gene could result in different isoforms and cause a range of accelerated ageing and peripheral nerve disorders, including Hutchinson-Gilford progeria syndrome and Charcot-Marie-Tooth disease [36]. VCAN and LAMP2 were reported with related changes in isoform ratios with advancing age [15,16]. VCAN, encoding chondroitin sulfate proteoglycan 2, was a key factor in cell adhesion, migration and inflammatory response [11,37]. Novel mutations and unbalanced alternative splicing were reported to associate with Wagner vitreoretinal degeneration [38]. LAMP2 encodes lysosome-associated membrane protein 2, one of the lysosome-associated membrane glycoproteins. Its deficiency resulting from splicing defects might lead to the Danon disease reported with clear degenerative features [39]. Moreover, some genes known to be spliced in cellular senescence were also identified. CD44, a senescence-induced cell adhesion gene [40], was identified with three differential splicing events in our work.

Our research elucidated that a group of RNA binding proteins were significantly down-expressed in cellular senescence. They played crucial roles in post-transcriptional processing of RNAs, especially in mRNA splicing. The disruptions to these RBP gene expressions in CS might lead to a global aberrant mRNA stability and differential splicing patterns. We took further study to perform an enrichment analysis of the RNA binding information within the differential AS event regions and to predict eight splicing regulatory RBPs. Apart from senescence-associated phenotypes, the detected RBPs’ importance has also been implicated in the process of aging and age-related degenerative diseases [41]. Three of them, namely, SRSF1, HNRNPK, HNRNPM, were characterized by their significant negative correlations with age in several population studies of human peripheral blood [16,42]. PTBP1 (down), in parallel with HNF4A (up), was identified as the most significant blood biomarkers in Parkinson’s Disease through transcriptomic and network-based meta-analysis [43]. In addition, there were twelve enriched RBPs without significantly differential expression. Two of them, namely, PCBP2 and HNRNPC, were differentially spliced but without alterations on gene expression levels, whose regulatory ability might change on the post-transcriptional levels.

We also looked into the expression alterations of eight predicted CS-associated RBPs in the quiescence condition. We collected two datasets of cell quiescence of the non-immortal cell lines (Table S1), namely, BJ and IMR90, and examined the differential expression levels (Table S11). Among the eight predicted RBPs, some were also down-regulated in quiescence. For example, SRSF1 and SRSF7 showed significant down-expression in both of two quiescent datasets, while PTBP1 and HNRNPM were identified down-regulated in only one dataset. We hypothesized that these RBPs down-regulated in both quiescence and senescence conditions might be associated with cell cycle arrest per se. Meanwhile, some other RBPs like HNRNPK, HNRNPUL1, RBFOX2 and QKI were not significantly changed in quiescence condition. They were likely to be associated with senescence-inducing changes of gene expression and to participate in senescence-specific biological processes. For example, QKI [30,31] and HNRNPK [35,44] were found to regulate SASP genes and to maintain telomere function, respectively.

In summary, our work provides systematically genome-wide evaluation of splicing alterations through an integrative meta-analysis and uncovers the splicing regulatory relationships with candidate RBPs during cellular senescence. Previous work showed that some of our identified splicing genes or RBPs inﬂuenced CS-associated phenotypes and multiple age-related diseases. Our finding is the first time to identify potential splicing regulatory RBPs and to build the main splicing regulatory modules of CS. The restrictions of our study include the unbalanced sample size of cell types and the data we used were only on the mRNA levels. Facing such an essential role for isoform splicing, the current challenge is to elucidate how specific splicing changes contribute to senescence with functional impact. The critical role of the upstream RBPs and their relationships with splicing events need to be investigated in the further studies.

## MATERIALS AND METHODS

Figure S3 shows the pipeline of data processing. The data sets and tools used in this study are presented in this section.

### Data collection

RNA-seq datasets in SRA format were collected from Gene Expression Omnibus (GEO) database [45] by searching with keywords “cellular senescence” and “senescent” (Table S1). Only datasets on non-immortal cell lines with at least two biological replicates and accompanied with information of senescence tests (such as growth curve and senescence-associated beta-galactosidase (SA beta-gal) activity) were remained. In total, we collected 51 senescent samples and 44 growing samples.

SRA data were converted into *fastq* format by using *fastq-dump* from SRA Toolkit 2.8.1. We performed individual differential analysis for consideration of different cell lines or induction methods in the following study. For example, as dataset SRP062872 contained two type of induction methods, we classified senescent samples into two groups, namely, RAS-induced senescence and drug-induced senescence. Then, we compared them with the growing samples respectively to perform the individual differential analysis.

We collected two RNA-seq datasets of quiescent samples from GEO database (Table S1). Only the datasets on non-immortal cell lines with at least two biological replicates were used in our analysis.

We collected 241 known human RBPs from ATtRACT [46] and ENCODE project (https://www.encodeproject.org/) [24,26]. 192 RBP genes with RNA binding information (either high confident motifs or eCLIP data) and expressed in at least one dataset were took into further study.

RBPs’ eCLIP-seq data (Table S12) and RNA-seq data (Table S13) treated with shRNA knockdown against single individual RBP (and control shRNA against no target) were downloaded from ENCODE [26].

### Differential expression analysis

Reads were mapped to human reference genome hg19 using tophat (v2.1.1) [47]. Raw read counts were calculated using HTSeq [48] for the following differential expression analysis.

Three methods were combined to make meta-analysis. First, we pooled all samples from ten datasets together and performed t-test to estimate the differential expressed levels of genes between senescent samples and growing ones. Second, differential expression analysis in each individual experiment was made via using DESeq2 and p-values in all comparison were combined through Fishers’ combining method. Third, inverse-normal method was used to combine p-values from the second method, weighing by sample size in each data set. We performed multiple test corrections in all three methods. For the first method, the significance threshold of p-value was calculated through a permutation procedure to control family-wise error rate (FWER) at 0.05. For each permuting step, we shuffled all sample labels randomly and generated p-values as described above. The smallest p-values from individual shuffling steps were remained to estimate the distribution under null hypothesis and were sorted increasingly. We set the 50th p-value as the threshold among the 1000 times of shuffling. For the other two methods, we adjusted p-value through Bonferroni correction and set the threshold as 0.01. Only genes that found significantly differentially expressed in all three approaches were defined as differentially expressed genes in CS.

To remove batch effect in the sample merging method, we normalized read counts of each data set separately through DESeq2 [49] and performed quantile normalization of the log-transformation of the normalized read counts across all. Genes with positive read counts in at least one sample were remained in the following analysis. In order to evaluate the success of batch effect removal in the method of pooling all samples together, growing samples were visualized in a Principal Component Analysis (PCA)-plot. The first component separated the samples according to tissue origins despite of datasets origins (Figure S2B). In the plot, samples were grouped into three clusters: foreskin fibroblasts (BJ and HFF), lung fibroblasts (IMR90 and WI38) and astrocytes. PCA of all samples revealed that both tissue origins and cellular states (senescence or growing) accounted for most variance (Figure S2C). PCA was performed using R-package labdsv [50].

We characterized the similarity between experiments by clustering normalized ranks of p-values. For each individual experiment, DESeq2 with one-sided test was used to detect differentially expressed levels of genes. Then, p-values from the one experiment were normalized through order statistics [51,52]. For the global comparison, only the top 2000 up-regulated/down-regulated genes in at least one individual analysis were used for the comparison (Figure S1B).

### GO enrichment analysis

Online tool DAVID was used to identify enriched Gene Ontology (GO) terms [53]. Due to the large number of GO terms significantly enriched with differentially expressed genes, it was necessary to cluster them to remove redundant ones. If two GOs shared similar set of genes, they might be related. *Kappa* statistics was used to measure the similarity between two any GO, which was just the same as in DAVID [54]. Any GO pair with *kappa* value more than 0.05 was classified as the same class. And for each class of GOs, only top three GOs are shown in Figure S1C and top two ones are shown in Figure 2C.

### Alternative splicing analysis

rMATs (v3.2.5) [19] is a hierarchical model to identify alternative splicing events with an associated change in exon inclusion levels, also known as Percent Spliced In (ΔPSI), from replicate RNA-seq data. We used rMATs to compare senescent samples with growing ones in each individual dataset. We ran it by using parameter c 0.0001 and reads mapped to the exon body as well as splicing junctions to detect the differences in exon inclusion levels. Splicing events with average combined read counts (inclusion plus skipping reads) in either senescent samples or growing samples less than five were filtered out. In each experiment, the splicing events with *fdr* adjusted p-value <0.1 and |ΔPSI|≥0.05 were detected differentially spliced. In order to identify consistently alternative splicing events in multiple datasets, both naive vote counting method and Fisher’s combined p-value method were applied in our study. Only events significantly differential splicing in more than three experiments and with combined *fdr* adjusted p-value <0.05 were defined as CS-associated differential splicing events.

### Motif enrichment analysis of alternative splicing event regions with RBPmap

In order to identify RBPs’ RNA binding sites that were significantly enriched in AS event regions, we collected known motifs from a RBP-motif database, ATtRACT [46] and performed motif scan by using a web server, RBPmap [55]. CS-associated differential splicing events were defined as Alternative Splicing Events (ASEs). Control events, also named as non-ASEs, with the same number of ASEs of each type were randomly selected from the background, namely, other not differential splicing events. We scanned every motif for its occurrence in both of the ASEs and non-ASEs with a p-value threshold at 0.05. Event regions were defined as the alternatively spliced exon body (defined as intronic regions that are 250 bp upstream or downstream of this exon), similar as previous research by Xing Lab [56]. 6 bp upstream of the 5’ splice site and 20 bp downstream of the 3’ splice site in intronic regions were excluded. We assigned each motif to its associated RBPs and performed enrichment analysis by comparing its frequency of occurrences in ASEs and non-ASEs via Fisher’s exact test (one-sided test). For the RBPs with multiple motifs, the one with smallest p-value was remained.

### Enrichment analysis of alternative splicing event regions with ENCODE eCLIP-seq data

The binding peaks with format of “bed narrowPeak”, defined by CLIPper [57], of eCLIP-seq data were extracted from ENCODE. First, only significantly enriched peaks, with fold enrichment ≥3 and p-value ≤10^-5^, were retained for the following analysis. Second, the ASEs and non-ASEs (defined in motif enrichment analysis section) were overlapped with filtering peak results respectively by using BEDTools [58]. Finally, two groups of events with or without high-confidence peaks were counted separately to determine the enrichment for each RBP via Fisher’s exact test (one-sided test). Fisher’s combining method was used to integrate the p-values of the enrichment result of motif scanning and eCLIP peaks to assess the RNA binding ability of each RBP. We applied *fdr* adjustment to the combined p-value and set the threshold as 0.05. Only the RBPs observed with significant q-values in both of two combination methods were defined as enriched ones.

### Validating the regulatory roles for the predicted CS-associated regulatory RBPs

We colleted 121 groups of RBP knockdown RNA-seq data fom ENCODE. For each group of RBP gene knockdown experiment, we also used rMATs to detect the differential splicing events between knockdown samples and controls with the same parameter as above. Threshold of *fdr* adjusted p-value and |ΔPSI| were set as 0.05 and 0.1 respectively. The enrichment analysis was performed on the knockdown induced differential splicing events and the CS-associated splicing events through Fisher’s exact test (Table S8). RBPs were ranked increasingly according to the p-value from the enrichment analysis. Then, pre-ranked gene set enrichment analysis tool GSEA [27] with weighted mode was used to validate enrichment of the pre-ranked RBP set and our predicted eight CS-associated splicing regulatory RBPs.

### SNV annotation

We used ANNOVAR [59] to map known SNVs to the CS-associated differential splicing event regions (as defined above). The associations between SNVs and phenotypes were annotated according to the NCBI ClinVar database [22].

### Constructing the splicing regulatory modules

First, we assigned the detected differential splicing events to the candidate splicing RBPs according to the corresponding RNA binding information, which was obtained through analysis of scanning motif and eCLIP peaks. For each candidate RBP, only differential splicing events with either significant enriched motifs or eCLIP peaks were taken as its targeting events. Then, GO enrichment analysis was performed for the RBPs’ targeting genes with splicing events respectively to infer their potential biological functions.

## Supplementary Material

Supplementary Figures

Supplementary Tables
