# Supplementary material for: Regulatory RNA binding proteins contribute to the transcriptome-wide splicing alterations in human cellular senescence
Source: Aging (Albany NY). 2018 Jun 24;10(6):1489–505. doi: 10.18632/aging.101485 (PMC6046225; doi:10.18632/aging.101485)
Supplement: Supplementary Figures [file aging-10-101485-s001.pdf]

SUPPLEMENTARY MATERIAL

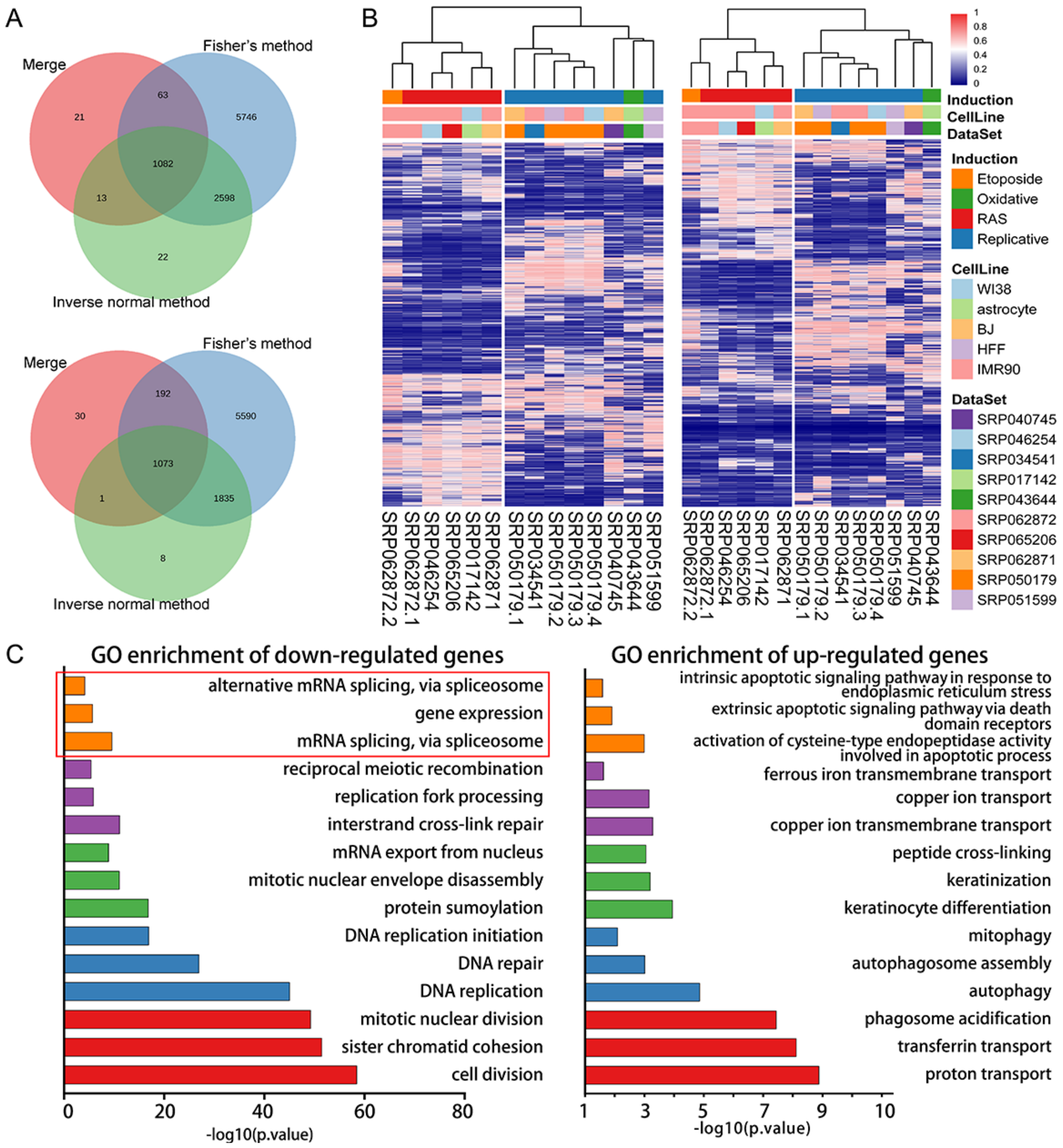

Figure S1. Differentially expressed results of cellular senescence. (**A**) The venn plot of the identified differentially expressed genes in three approaches. (**B**) Heatmap of genes with one vote in at least one experiment. Down-regulated results (left); up-regulated results (right). Heat map was used a rank-based visualization method to present the differential expression levels of genes ranked top 2000 down/up regulated genes in at least one dataset. Each column represents an experiment and each row represents one gene. A normalized rank transform is performed on each individual experiment by sorting the p-values from the smallest with the lowest 0 (blue) to the largest with the highest 1 (red). (**C**) GO enrichment of DE genes, we clustering the GO terms with the intersection of genes within pairwise GO terms. For the each of clustering, we only showed the top three GOs. Enriched GO of down-regulated genes (left); enriched GO of up-regulated genes (right).

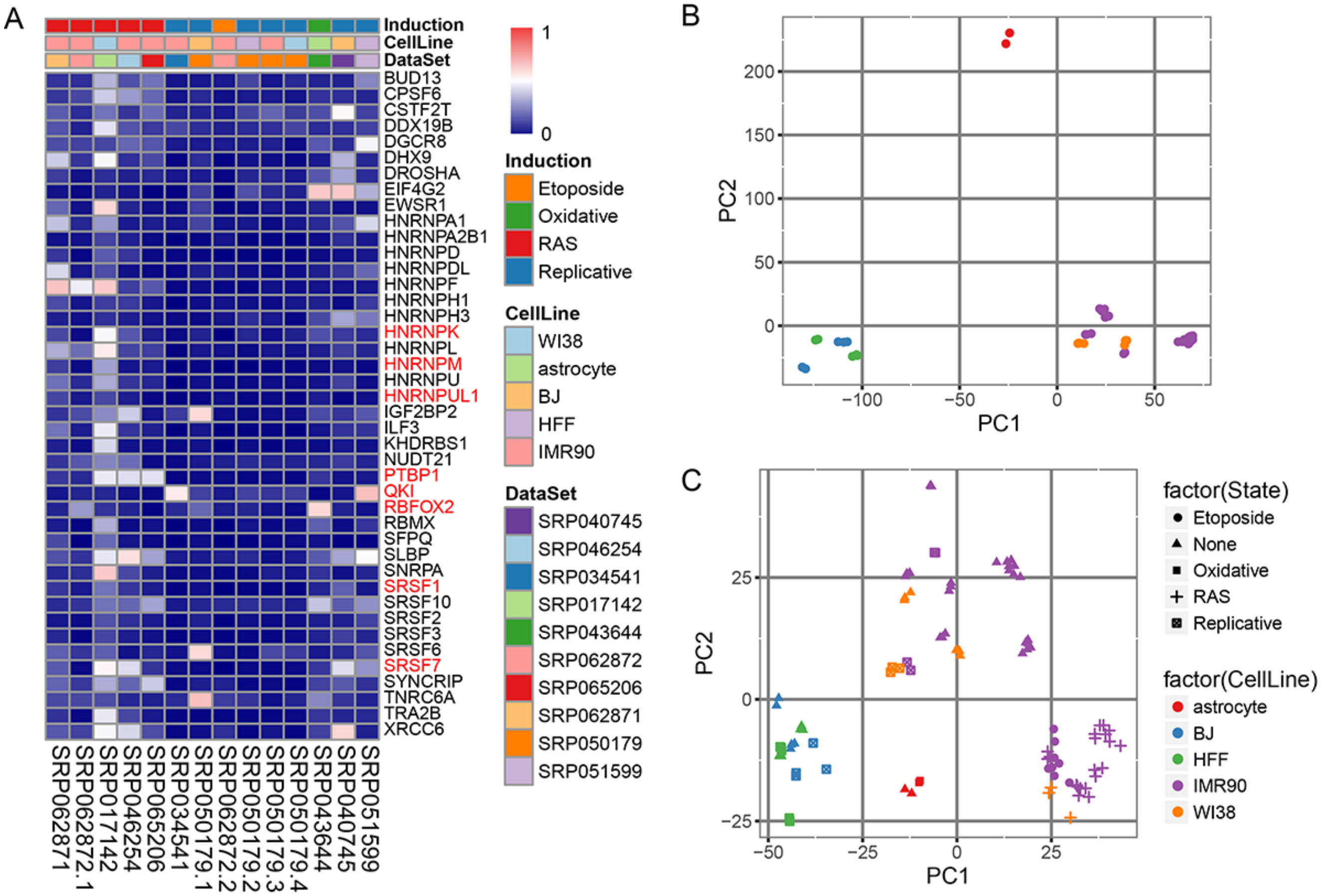

Figure S2. Differentially expressed results of cellular senescence. (**A**) Differential expression levels of the identified down-regulated RBPs. (**B,C**) PCA plot of growing samples and all samples with read counts. PCA plot of all growing samples (B). The samples were separated according to the tissue origins. PCA plot of all samples together (C). The main variance is associated with tissues and cell state (senescent or growing).

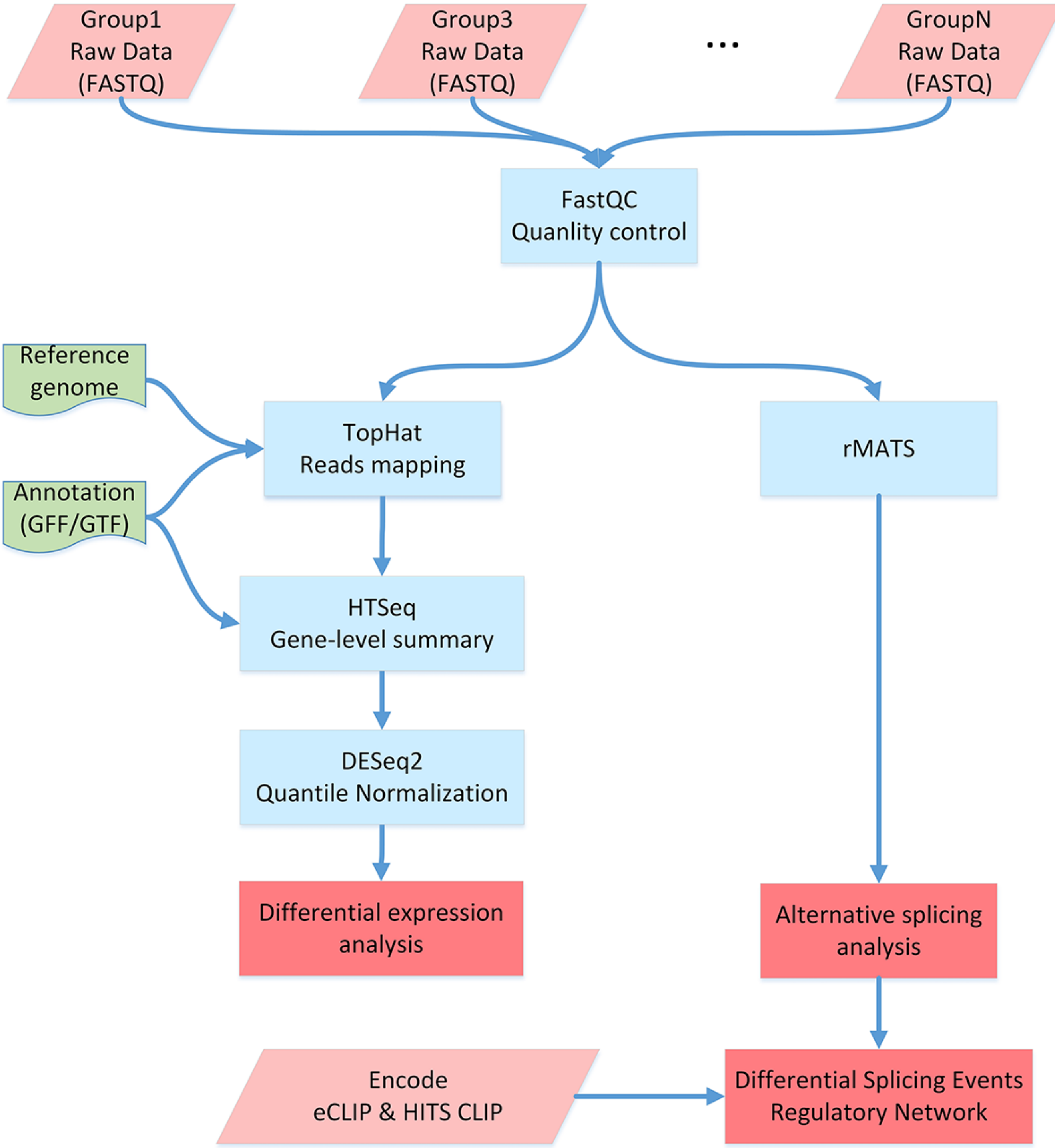

Figure S3. Processing pipeline of our research. It provides the main tools and analysis structure in our differential expression analysis and differential splicing events analysis.

Please browse the Full Text version to see the data of **Supplementary Tables**

Table S1 Collected RNA-seq datasets of cellular senescence and quiescence in detail

Table S2, 3 DE gene list (up, down)

Table S4 All GO enrichment results of differentially expressed genes (up, down)

Table S5 Differential expression levels for RBPs with RNA binding information

Table S6 The statistics of the identified ASEs from each dataset

Table S7 Meta-analysis result of splicing events identified in multiple experiments

Table S8 Enrichment results of RNA binding information

Table S9 Enrichment results of the single RBP knockdown experiments

Table S10 Phenotype-associated SNVs within the CS-associated differential splicing event regions

Table S11 Differential expression levels of predicted eight RBPs in quiescence

Table S12 eCLIP data collected from *ENCODE*

Table S13 Single RBP knockdown RNA-seq data collected from *ENCODE*
